# Notes and News

**Published:** 1893-09-02

**Authors:** 


### Notes and News.

Collected and Communicated.

A quarterly General Court of the Governors will be held at the London Hospital on Wednesday, September 6th, at two o'clock, to receive the report of the House Committee for the last three months.

Splendid weather favoured the Hospital Sports held at the Paddington Recreation Grounds on Saturday last. Fully 5,000 spectators were present, and the races evoked much enthusiasm. The prizes were presented by Sir John Astley.

Sympathy in illness is a quality we all require, and above all, we demand it from our medical advisers. If a doctor can say, "I know from experience just what you are feeling," we are far more inclined to trust to his prescription. A story is told from the East of a certain man who fell from a house-top and broke a rib. The doctor was sent for. On arriving, the patient inquired, "Hakim, have you ever fallen from a roof and broken a rib?" The doctor replied he had not. "Then go away at once, please," was the reply. "I want a doctor who has fallen from a roof, and knows what it is."

The Recreative Evening Schools Association has a most useful future before it. The main object, *i.e.*, the continuation of the education commenced at the elementary schools, is supplemented by social advantages offered by the society also. These comprise homes for working girls and social institutes for working men and boys. The progress made by the association is quite astonishing, and shows that the appreciation of the advantages offered is very real In 1885 the number of schools was 839, with 24,232 average attendances; in 1892 the schools had multiplied to 1,604, with 65,561 attendances. The sphere of the society is ever increasing, and as it supplies a genuine want it is to be hoped that the donations and subscriptions upon which it is entirely dependent will increase in equal proportion. The offices are at 37, Norfolk Street, Strand.

We understand that the Directors of the Leith Hospital contemplate erecting new buildings, to contain additional beds, although those at present available are sufficient for the requirements of the population. Our attention has been called to the insanitary condition of the existing buildings in many respects, and also to the defective accommodation provided for the resident staff. We purpose sending our Commissioner to inspect the institution on an early date, and meanwhile we hope that the Directors will devote themselves to the urgent work of putting the existing buildings and defective accommodation into thorough order before they consent to expend money upon additional wards.

The new wing of the Poplar Hospital will be opened by the Right Hon. Lord Knutsford and Lady Knutsford on September 4th, at five p.m. The Hon. S. Holland has been a tower of strength to this institution, and has raised £22,500 of the £25,000 expended upon the new buildings and reconstruction works. This has been done without any fuss in rather more than two years, and is an object lesson which we hope many of the sons of influential men who have leisure will take to heart, and act upon in the best interests of our medical charities. We would suggest that the Committee of the Poplar Hospital should present the Hon. S. Holland with his portrait, and that it should be placed in the new Board-room for the encouragement of all who come after.

May the excellent example set by "Job-Master," whose letter was recently published in the *Times*, be followed by others of his kind. His customers having, many of them, left town, it is his intention for the next few weeks to make use of his carriages and horses for the benefit of the pale-faced London children, and to give them a breath of fresh country air by taking them for drives. He calls upon charitably-disposed people within five and ten miles of town to help him by providing tea for the children in their gardens or kitchens, and to communicate with him to that end. His name is not given, but all letters should be addressed to "C. C., care of Gastrell's Theatrical Booking Office, Sussex Place, S.W." It is pleasant to think of the healthy pleasure which will thus be given to some of the little ones into whose lives so small a portion of enjoyment finds its way, and we sincerely hope "Job-Master's" kind thoughtfulness will meet with a hearty response from those who can help him so materially in his plan.

Sir Henry Fletcher, Chairman of the East Preston Union, has issued an appeal for help to enable the unfortunate inhabitants of Worthing to recover in some measure from the terrible misfortune which has befallen them. A sum of nearly £1,000 has been collected locally, but this is now all but exhausted, and more funds are urgently need on behalf of the convalescent patients. As Sir Henry Fletcher justly remarks, convalescence from typhoid is exceptionally lengthy and wearisome, and the patients (there are some 500 cases now under treatment) are mostly poor people, who are quite unable to afford that care which is absolutely necessary if they are in any way to regain their health. It is proposed to provide homes where the worst cases may be treated, and to supply such luxuries as may be possible for those who can be left in their own homes. Seeing how money has lately poured in for the "Victoria" Fund, it is not too much to hope that substantial help will also be forthcoming in this instance also, where distress is quite as real and pressing, if not as sensational.

